# New non-randomised model to assess the prevalence of discriminating behaviour: a pilot study on mephedrone

**DOI:** 10.1186/1747-597X-6-20

**Published:** 2011-08-03

**Authors:** Andrea Petróczi, Tamás Nepusz, Paul Cross, Helen Taft, Syeda Shah, Nawed Deshmukh, Jay Schaffer, Maryann Shane, Christiana Adesanwo, James Barker, Declan P Naughton

**Affiliations:** 1 School of Life Sciences, Kingston University, UK; 2 Department of Psychology, University of Sheffield, UK; 3 Department of Biological Physics, Eötvös Loránd University, Hungary; 4 School of Pharmacy and Chemistry, Kingston University, UK; 5 School of the Environment, Natural Resources and Geography, Bangor University, UK; 6 Applied Statistics and Research Methods, University of Northern Colorado, USA

**Keywords:** random response technique, non-random model, Mephedrone, survey, illicit substances, epidemiology

## Abstract

**Background:**

An advantage of randomised response and non-randomised models investigating sensitive issues arises from the characteristic that individual answers about discriminating behaviour cannot be linked to the individuals. This study proposed a new fuzzy response model coined '*Single Sample Count*' (SSC) to estimate prevalence of discriminating or embarrassing behaviour in epidemiologic studies.

**Methods:**

The SSC was tested and compared to the established Forced Response (FR) model estimating Mephedrone use. Estimations from both SSC and FR were then corroborated with qualitative hair screening data. Volunteers (n = 318, mean age = 22.69 ± 5.87, 59.1% male) in a rural area in north Wales and a metropolitan area in England completed a questionnaire containing the SSC and FR in alternating order, and four questions canvassing opinions and beliefs regarding Mephedrone. Hair samples were screened for Mephedrone using a qualitative Liquid Chromatography-Mass Spectrometry method.

**Results:**

The SSC algorithm improves upon the existing item count techniques by utilizing known population distributions and embeds the sensitive question among four unrelated innocuous questions with binomial distribution. Respondents are only asked to indicate *how many *without revealing *which *ones are true. The two probability models yielded similar estimates with the FR being between 2.6% - 15.0%; whereas the new SSC ranged between 0% - 10%. The six positive hair samples indicated that the prevalence rate in the sample was at least 4%. The close proximity of these estimates provides evidence to support the validity of the new SSC model. Using simulations, the recommended sample sizes as the function of the statistical power and expected prevalence rate were calculated.

**Conclusion:**

The main advantages of the SSC over other indirect methods are: simple administration, completion and calculation, maximum use of the data and good face validity for all respondents. Owing to the key feature that respondents are not required to answer the sensitive question directly, coupled with the absence of forced response or obvious self-protective response strategy, the SSC has the potential to cut across self-protective barriers more effectively than other estimation models. This elegantly simple, quick and effective method can be successfully employed in public health research investigating compromising behaviours.

## Background

Outcome based evaluation of interventions, which play a central role in public health prevention, need to show the effect the policy or intervention makes at the public level. Whilst a plethora of literature focuses on evaluating various social marketing campaigns that tackle public health and safety issues such as drug use, health compromising lifestyle choices, unprotected or risky sexual behaviour, or unsafe driving practices, tend to rely on self reports, regardless of whether or not they were conducted in laboratory or field settings [1-3]. The issues that may hinder an evaluation of any health promotion [4] are further complicated by the influence of social desirability that may cast doubt over the validity of self-reported information when to the study topic relates to socially sensitive behaviour [5]. In addition to public health concerns where obtaining accurate information on drug use is vital in establishing the need for and to evaluate preventive measures or intervention strategies, policy makers in public service utilities and law enforcement agencies also require the most accurate estimates of the problematic behavioural choices as possible in order to make informed choices.

The need to obtain the maximum intelligence on health related behaviours stems from the necessity to develop and deploy optimal intervention measures to counteract consistent failures to attain acceptable levels of behaviour across a wide range of health practices. These range from adherence to medication, resistance to addiction, avoidance of exploration of social drug use through to uptake of illegal and health damaging performance enhancement agents. The immense health, financial and social consequences of enhancing these health related behaviours has led to decades of investigation into improved approaches to obtain accurate data on sensitive personal behaviours.

Investigating the epidemiology of socially sensitive or transgressive behaviours such as illicit drug use, unhealthy weight management practices, risky behaviour, cheating, doping or non-adherence to prescribed medication or treatment, is hindered by respondents evasively answering questions about sensitive behaviours [6]. A recent research programme provides further evidence for self-protective strategic responding, even under anonymous answer conditions [7-9]. Consequently, much effort has been made to develop reliable methods to collect valid epidemiological data in these sensitive behavioural domains.

Approaches range from techniques such as the Bogus Pipeline [10] to providing incentives for honest answers such as the Bayesian Truth Serum (BTS) [11]. Whilst the Bogus Pipeline has been used for decades and accumulated reasonable evidence that the BPL shifts self-reports toward veracity [12], the BTS approach is relatively new and in need for further refinements [13,14]. Based on empirical evidence, Barrage and Lee [13] also suggest that to be effective, respondents may need to have a positive experience with and trust in the BTS method, which can lead to respondents learning how to maximise their incentives and therefore their answers might be biased towards maximum income at the expense of telling the truth. Although these methods possess the potential to overcome to an extent, self-protective response bias by either evoking fear of exposure of lying or providing financial gain for truthfulness, their feasibility in self-administered epidemiological scale studies appears to be compromised. An alternative approach has made notable progress in collecting data on sensitive behaviours through the development of indirect methods using randomisation or deliberate uncertainty to provide respondent protection over and above ensuring anonymity [6].

The concept behind randomised response models (collectively termed RRT) rests on introducing a randomising element to the survey question by using some device (e.g. by rolling a dice, flipping a coin or picking a card) which determines how the respondent should answer [15]. Since the researcher has no control over this randomising device, answers cannot be directly traced back to any particular individual, which in turn heightens the respondents' sense of increased protection. A common characteristic of RRTs is that to obtain useful data on the sensitive question, the technique requires respondents to answer directly, in some form, the sensitive question. By contrast, non-random models (NRM) do not require a direct answer as they rely on implicit uncertainty rendering impossible the link between an individual and the sensitive behaviour. Whilst NRMs build on combining the sensitive question with unrelated innocuous questions, some RRTs also incorporate innocuous questions where the population prevalence may or may not be known. When population prevalence needs to be established, it requires an independent sample randomly selected from the same population.

### Randomised response models

The RRT aims to elicit sensitive, embarrassing or compromising information that may portray respondents unfavourably. The common characteristic of the RRT is that sensitive behaviour estimation can only be made at the aggregated population level. The method is based on the principle introduced by Warner [16] using a spinner as a randomising device to gauge the proportion of the sample with a compromising behaviour. The method assumed that any person in a sample is either characterised by the behaviour (group A) or is not (group B). The respondents, hidden from the interviewer, were asked to use the spinner which either landed on group A or on group B and answer with a simple 'yes' or 'no' depending on whether the spinner pointed to the group he/she belonged to. Whilst the outcome of the spinner exercise for each individual was not known to the interviewer, hence protecting the individual, the chance that a spinner points to group A or B was known (*p *and *1-p*). Thus compared to the observed pattern of 'yes' and 'no' answers Warner was able to determine the proportion of respondents in the sample admitting the sensitive behaviour.

Subsequent adaptations of the RRT have covered a wide range of sensitive issues along with numerous attempts to refine the approach [15]. Among the wide array of models, the Forced Alternative/Response model, used only when the sensitive question is presented [16], has been found to be one of the most efficient variants of Warner's original conception [17]. Recently the RRT method has been expanded to multi-item scales and tested with male date rape attitude [18] and alcohol abuse [19]. The extension of the RRT to multi-item scales allows its application to psychological measures such as attitudes toward sensitive issues. This approach can be expanded to areas where honest responding might be compromised by self-protective lying, for example illegal substance dependence, domestic violence, disordered eating or cheating and doping use in sport.

### Non-randomised methods

Research has shown that whilst respondents understand the reason behind the use of the RRT approach in surveys, they generally find it obtrusive and favour simpler approaches [20]. Contrary to the RRT, non-random models present a more straightforward approach that provides protection by asking the number or combination of behaviours respondents are engaged in rather than asking about each behaviour in turn.

The non-randomised model (NRM) has received increased attention lately. A recent review [21] showed that NRMs appear to successfully address many of the limitations typically associated with RRTs such as the need for a randomisation device which often requires interviewers; forcing participants to say 'yes' to an embarrassing question when their honest answer would be the opposite or requiring a direct answer to the same question. Contrary to the RRT, in the NRM every participant is required to answer the research question in an evasive way. The fact that a response is required to the research question can help participants to feel that they have made a contribution by volunteering to take part in the research whereas with many RRT variations, a significant proportion of respondents are simply instructed to ignore the research question and just say 'yes' or 'no'. Owing to this characteristic, NRMs can also be more efficient with comparable or even increased privacy protection levels.

Alternative approaches have been progressively developed which preclude the need for the randomising device. These include an item count method [22], later termed the 'item count technique' [19] and later the 'unmatched count technique' [23]. In a similar concept to the unrelated question (UQ) method [16,24], item counts (IC) utilise a simple response task whilst embedding the sensitive question in a list of innocuous questions. In place of the randomising device the experimental group receives all questions with instruction and are asked to indicate only the number of affirmative answers. As a control sample is required to establish the population prevalence of the innocuous questions, respondents are randomly assigned to one of two groups (experimental and control), where the control sample receives the identical list of questions minus the sensitive question. The mean number of 'yes' responses are compared between the two groups. Assuming that the innocuous behaviour is equally manifest in both groups, the difference between the observed proportion of 'yes' answers must be due to the presence of the sensitive question in one of the groups and not the other.

Using prior knowledge of the population prevalence for an innocuous question, has led to the development of a number of competing techniques over the past five years. In these models, the innocuous question is outside the researcher's control, independent of the research question but the population prevalence is already established such as birth month or season, geographical location for the person or a family member. The Triangular Model (TM) and the Crosswise Model (CWM) use a combination of a sensitive and an innocuous question with known population prevalence [25]. The question and answer options are then placed in a 2 × 2 contingency table where two 'quadrants' relate to the innocuous questions are with known population prevalence (e.g. 3/12 and 9/12 if someone's birth month is used as the innocuous question). The other two quadrants represent the binomial response options to the sensitive question. In the TM respondents are asked to indicate whether they belong to the No-No quadrant or any of the other three quadrants (Yes-No, Yes-Yes or No-Yes). The CWM asks people to indicate whether they belong to any of the mixed categories (Yes-No and No-Yes) which only reveals that *one of the two *statements is true but *which *one remains hidden. Similarly, the Hidden Sensitivity (HS) model for two sensitive questions with binary outcomes using one quadrant such as season for birthday or geographic location (e.g. South/West/North/East, East/West side of a river or any criteria that creates meaningful and useable groups) [26]. In this technique two response pathways are provided. Respondents are required to either answer truthfully or are forced to an option for the non-sensitive question (e.g. about birth date or place of living) based on their answers to the two sensitive behaviours. The drawback of this technique is that only those who belong to the category of not having a sensitive behaviour (0,0) are asked to answer the innocuous question honestly, whereas others (0,1; 1,0; 1,1) are forced to select an answer for the innocuous question based on their sensitive behaviour. Therefore, people admitting to a sensitive behaviour (or both) are protected by the true answers of those who do not have a sensitive behaviour to declare. The advantage of the HS model over the Triangular or Crosswise models is that HS allows two sensitive questions to be simultaneously investigated [27].

Other models such as the Unmatched Count Technique (UCT) [28] or the Cross-Based Method (CBM) and the Double Cross-based Method (DCBM) [29] work with unknown population prevalence. The common characteristic of these models is that an independent sample randomly drawn from the same population is required to establish the prevalence rate for the innocuous questions in order to estimate the prevalence rate for the sensitive question. The UCT [28] contains two parallel questionnaires with several innocuous questions but only one version of the questionnaire features the sensitive questions. The total number of endorsed answers is calculated for each version independently, and then compared. The difference between the two sample means indicates the proportion of the respondents who endorsed the sensitive question.

Currently, studies comparing the performance of the item count method to other NRM or RRT models, or direct self-reports, are inconclusive. Coutts and Jann [28] found that the UCT outperformed the RRT counterparts in assessing many sensitive behavioural domains. By contrast, Tsuchiya et al. [30], using a web-based survey, compared the item counts to direct self reports and concluded the item count technique yielded lower numbers of endorsed behaviour. However, Tsuchiya's [30] list of behaviours contained items to which over-reporting can reasonably be expected (e.g. donating blood), which might have skewed upwards the total numbers of reported behaviours in direct self-reports. Where differences were found between self-reports and item counts (using CBM and shoplifting) the differences were explained by the sample demographic. The largest difference was found among the middle-aged, domiciled in urban areas and highly-educated (e.g. in or completed tertiary education) female respondents [30].

#### Practical issues

Constraints of each approach were associated with whether or not the population prevalence used for the non-sensitive questions was known. When this information is not available, the research requires an independent sample of significant size to establish this, parallel to collecting a sample to answer the research question about some sensitive issue. Furthermore, the chosen probability that requires respondents to answer truthfully determines the proportion of the sample that is directly useable to answer the research question. Finally, the actual prevalence rate of the target behaviour also has an effect on the minimum required sample size.

Investigating the efficiency of the RRT, Lensvelt-Mulders et al. [17] compared five RRT methods and found the Forced Response method and a special from of the Unrelated Question design the most efficient requiring about 2.2 times the sample size required of a direct self-report method. Sample sizes for the Crosswise model were estimated for a number of combinations of power and population prevalence [31] where estimates for minimum required sample sizes ranged between 2.5 and 19.3 times the sample size required for direct questioning surveys. Based on these simulations, the Crosswise model's efficiency compared favourably to Warner's [32] model.

An alternative way to think about efficiency is to consider the proportion of the population sample solely used to provide an estimate of the population prevalence for the non-sensitive questions. This 'waste', which accompanies most models, is the acceptable efficiency cost of providing the added anonymity. The proportion of the sample inefficiency ranges between 25% and 75%, depending on the research design. Consequently, in order to achieve a sample size with sufficient statistical power for meaningful analysis there is a requirement for more extensive data collection than in a typical survey.

### Aims

The recent change in legal status (in the UK) of the drug Mephedrone provided an opportunity to explore a novel approach to data collection on a sensitive issue. Mephedrone is a central nervous system stimulant that produces effects similar to amphetamines. It produces a euphoric effect, and has been reported to increase empathy, stimulation and mental clarity, but can lead to adverse effects such as nasal irritation, tachycardia and restlessness [33]. Although limiting in scope (i.e. we asked about the use of one specific drug), Mephedrone was a topical choice at the time of the study's conception as it had been reclassified as a Schedule 1 Class B drug on April 16^th ^2010 [34], making it unlawful to possess, produce, and/or distribute without licence and carrying a five year prison sentence for possession and up to 14 years for producing, selling or distributing. The ban generated considerable debate, with some expressing discontent about the hastened reaction and the generic ban [35] along with a concern that the ban may not stop Mephedrone use, but could make the demand and supply clandestine, leading to unintended consequences from the addition of toxic excipients (through "cutting" or chemical by-products) and thus present an even greater danger to health [36]. In spite of the new legislation, internet retailers appear to have continued to sell products under different brand names that contain, albeit unlabelled, Mephedrone-like substances [37]. This case is a good illustration of the situation when the change in regulation could (and should) have been supported with at least an estimation of what proportion of the population uses Mephedrone and is at risk.

Recent inter-disciplinary approaches to estimating doping prevalence in sporting sub-populations has led to advances in estimation through improved efficiencies [38]. The current study aimed to develop and test a new research tool for use at the epidemiological scale. To achieve this aim, a fuzzy response model, Single Sample Count (SSC), was proposed.

## Methods

The study utilised a mixed design questionnaire method with chemical analysis of hair samples collected from the questionnaire respondents. This approach has been successfully employed in research investigating social cognitive factors in prohibited performance enhancing and illicit drug use [7,8].

To establish validity and reliability, the SSC was compared to an established RRT model, the Forced Response (FR), estimating Mephedrone use in a three-month period preceding the data collection. Estimations from both SSC and FR were then corroborated with qualitative hair analysis. Ethical approval was obtained from the two HEIs' Research Ethics Committees. Data were collected in two sites: a rural area in north Wales (51.3% of the surveys; 92.8% of the hair samples) and a metropolitan area in England (48.7%) from 318 volunteers (mean age 22.69 ± 5.87, 59.1% male). Of the 153 hair samples, 95 (61.7%) were donated by males. The majority of the data (91.5% of the questionnaires and 92.2% of the hair samples) were collected in May-June 2010, capturing the period in which Mephedrone has become a controlled substance in the UK. The remaining samples were collected up to February 2011.

### Measures

Along with the newly developed SSC, the questionnaire consisted of an established RRT, the Forced Response model [16], incorporated into the questionnaire in alternating order to mitigate any potential learning or priming effect, and always separated by four single questions evaluating the respondents' understandings and social projection of Mephedrone among a student population. To establish prevalence of recent use, the sensitive question asked respondents to indicate whether they have used Mephedrone in the last three months. One-hundred and fifty-three (48.43%) of the questionnaire respondents were asked to provide a hair sample for Mephedrone analysis to determine the drug's use over approximately three months prior to the study survey.

#### Forced Response model

The FR method has been shown to be one of the most efficient designs [15,17] and was consequently considered suitable as a validation tool for the new method. This variation of the FR [39] requires a pair of ordinary D6 dice. Respondents were instructed to shake the die in an opaque container in order to hide the score from all other observers and then to answer the following question 'Have you used Mephedrone in the previous three months? (Yes/No)' according to the outcome. If the combined score from the two dice is

• 2 - 4 = ignore the question and tick the 'Yes'

• 11, 12 = ignore the question and tick 'No'

• 5-10 = answer the question truthfully by ticking either 'Yes' or 'No'

As for scores 5-10 there are more variations (27/36) for suitable dice outcome than for scores 2-4 (6/36) or 11 and 12 (3/36), theoretically 75% of the respondents were instructed, by chance, to answer the target research question honestly.

#### Additional questions

In addition, three questions were included to gauge directly reported opinion, belief about health hazards and social projection. The questions were:

• In your opinion, should Mephedrone be a controlled substance? (Yes/No)

• What percentage of students in the UK do you think use Mephedrone (0% = nobody, 100% = everybody)? (Yes/No)

• On a scale of 1 (not harmful at all) to 10 (very harmful), how harmful do you think Mephedrone is for your health?

These questions were also used to establish that the two samples collected at different locations differed significantly.

### Analyses

#### Statistical analyses

Prevalence rates for the last three months were estimated using model specific formulae (detailed below). Testing sample means against the pre-set value was performed using single sample t-tests. The 95%CI for the binomial distribution was calculated using the Wilson interval. Simulations for establishing the required sample size for the SSC model were performed using varying levels of prevalence rates. Statistical analyses were performed using PASW 18.0, R and Minitab.

#### Hair analysis

Hair samples were screened for the presence of Mephedrone using a qualitative method developed in-house, Liquid Chromatography-Tandem Mass Spectrometry (LC-MS/MS). Sample preparation included enzymatic digestion to preserve the drug and liquid-liquid extraction as detailed below. All solvents/chemicals apart from Mephedrone and Mephedrone -d3 were of analytical or general purpose reagent grade and purchased from Sigma-Aldrich UK Ltd (Gillingham, Dorset, UK). Mephedrone and Mephedrone-d3 were purchased from LGC Ltd, (Teddington, Middlesex, UK). Mephedrone-d3, the triply deuterated form is used as a standard reference for mass spectrometric measurements.

##### Hair digestion

Hair (50 mg) was cut into fine segments and Cleland's Reagent (100 mg) was added followed by the addition of the enzyme Proteinase K (15 mg). Internal standard Mephedrone-d3 (100 μL) with 5 ng total concentration was added to the mixture and finally incubated with Tris buffer (1 mL) for 2 hours at 37.5°C with constant stirring.

##### Liquid Liquid Extraction (LLE)

The digested hair solution was then placed in a centrifuge tube for Liquid-liquid extraction with hexane (3 mL). The contents of the tube were mixed using a vortex mixer and centrifuged for 5 min at 1750 × g. The top layer was decanted using Pasteur pipettes and placed in a glass test tube. The extracted samples were dried completely with nitrogen gas and reconstituted with 100 μL acetonitrile.

##### Qualitative analysis

Qualitative analysis was carried out using a Thermoscientific liquid chromatography - tandem mass spectrometry (LC-MS/MS) system (Fisher Scientific UK Ltd, Loughborough, Leicestershire, UK). Three microlitres of reconstituted sample solution were injected into an Agilent SB-C18 column (Agilent Technologies UK Ltd, Wokingham, Berkshire, UK), (maintained at 45°C) for analysis. Acetonitrile (with 0.1% v/v formic acid) and water were used as mobile phase solvents. Total flow rate through the column was 200 μL/min. The LC mobile phase gradient composition is detailed in Table 1.

**Table 1:** LC-MS Methods for Mephedrone-d3

LC run time (min)		Acetonitrile in presence of (0.1% Formic acid)		Water (%)
0		60		40
3		100		0
4		100		0
5		60		40
10		60		40

**Retention time (min)**	**Lower Limit of Detection (ng)**	**Flow rate (μL/min)**	**Injection volume (μL)**	**Column Temperature (°C)**
1.92	0.5	200	3.0	45

The mass spectrometer was operated in selective reaction monitoring (SRM) mode to confirm the presence of Mephedrone. One precursor > two product ion transitions for Mephedrone (m/z = 178.1 > 160.1, 145.1) and Mephedrone-d3 (m/z = 181.2 > 163.2, 148.2) were monitored for qualitative analysis. The retention times for Mephedrone and Mephedrone-d3 were found to be 1.68 and 1.92 minutes, respectively. The calibration curve of Mephedrone was found to be linear in the range 1 ng/mL to 80 ng/mL (Lower limit of detection 0.5 ng/mL). Qualitative analysis of 154 hair samples was carried out using this calibration curve. Blank (control) hair without any Mephedrone was analysed to detect any artefact peaks that might elute at the same retention time or have similar isobaric transitions and thus lead to false results. However, no such interferences were observed. Thus, retention time and the most abundant SRM transitions were used to qualitatively determine the presence of Mephedrone.

### Sampling

Respondents completed either version of the question in randomly allocated order, separated by three questions soliciting responses to social projection and opinions to the target drug. Participants were recruited at universities and social spaces such as clubs and sport grounds outside the higher education institutions in the UK.

Respondents were approached by a data collector (two in total, one in each study region). The participation was voluntary. Participants who provided hair samples received a small monetary compensation (value of £5) for any inconvenience incurred in completing the survey. A hair sample was requested from each respondent upon completion of the questionnaire survey. Approximately half of the respondents provided usable hair samples. The exclusion criteria included treated (e.g. dyed or permed) or too short hair (less than 3 cm). Over 80% of the hair samples were dark in colour. The different sample sizes are owing to 70 volunteers receiving a 4-question (four innocuous questions only) version for the Single Sample Count (data not shown).

## Results

### Results from the survey

Using the full dataset (n = 318), no gender*region interaction effect was observed in social projection (F(1,310) = 1.547, p = 0.211; partial eta^2 ^= 0.004) or in perceived harm (F(1,308) = 1.242, p = 0.266; partial eta^2 ^= 0.005). Participants in the metropolitan area gave significantly higher estimates for others using Mephedrone (F(1,310) = 16.90, p < 0.001) but no difference was evidenced by gender (F(1,310) = 0.506, p = 0.478; Cohen's d = 0.100). The main effect for gender and region in perceived harm was significant (F(1,308) = 5.237, p = 0.023; F(1,308) = 5.000, p = 0.026, respectively). The slight discrepancies in sample sizes are due to missing values. Means and standard deviations by area and gender are shown in Table 2. The opinion regarding the legal status of Mephedrone overwhelmingly favoured control (81.7%), independent of area (Fisher's Exact Test = 2.104, p = 0.370) but not of gender (Fisher's Exact Test = 7.731, p = 0.011), with the preference for non-control of Mephedrone being higher amongst males (21.8%), compared to 11.6% amongst females.

**Table 2:** Social projection (0: nobody - 100%: everybody) and perceived harm (1: not harmful at all - 10: very harmful)

		Area		
		
		Rural	Metropolitan	ALL
Social projection	Male	28.00 ± 23.690	35.51 ± 23.231	31.45 ± 23.717
	Female	26.56 ± 20.780	40.68 ± 22.898	33.79 ± 22.926
	ALL	27.45 ± 22.572	37.74 ± 23.155	

Health risk	Male	5.87 ± 2.415	6.71 ± 1.912	6.26 ± 2.139
	Female	6.73 ± 1.968	7.01 ± 2.303	6.87 ± 2.139
	ALL	6.20 ± 2.286	6.84 ± 2.083	

Higher estimation of prevalence by participants in the metropolitan area is likely to be due to them holding different descriptive norms arising from the person's social context. Declared drug use among the active population (16-59) in England and Wales is consistently around twice as high in males than females and higher prevalence rates have been documented for urban compared to rural areas in last year's usage; with a similar but slightly more ambiguous trend for the 16-24 age group [40-42]. Biased social projection is one of the most intriguing areas in social cognition research. On the one hand, it suggests that the repeatedly observed association between self-reported behaviour or personality characteristics is explained by an egocentric bias (i.e. finding comfort in false consensus) [43], which is in keeping with the Bayesian approach [11]. On the other hand, particularly regarding the chosen sensitive and/or transgressive behaviours, it is suggested that the distorted perception of what eventually leads to a behavioural choice is congruent with this perception [44,45]. Conversely, recent research provides evidence showing that the prediction of population prevalence relates to the behaviour or characteristics the respondents wish to project about themselves, but not the actual behaviour [46,47].

Age was significantly negatively related, with the prevalence estimate (Spearman's r = -.150, p = 0.01) suggesting that younger people consider Mephedrone to be more prevalent. This is in line with the notion that Mephedrone is a drug for the young [33]. The correlation between age and the belief that Mephedrone was harmful was positive and significant (Spearman's r = .190, p = 0.001). As regional differences were not significant, the data from the two collection sites was combined and treated as one unified sample for future analyses.

### Estimation using the Forced Response model

Subsequent to completing the questionnaire, the prevalence rate for Mephedrone use, using the formula suggested by Tourengeau & Yan [6] was calculated as follows:

where:

π_1 _= probability that the respondent is forced to say 'yes'

π_2 _= probability that the respondent is forced to answer a sensitive question honestly

λ = observed percent that responded 'yes'

From the dice instructions, we see that *π_1 _*= 1 out of 6 and *π_2 _*= 3 out of 4. There were 74 'yes' responses out of 318 total, thus

The estimated prevalence rate for Mephedrone is 8.81%. The variance and standard error of this estimator are calculated as:

A 95% CI for the prevalence rate of Mephedrone would be the estimated prevalence rate ± the product of the z_α/2 _value and the standard error: 1.96 × 0.034159 = 0.061925, yielding the 95% CI of 0.026175 and 0.150025. Thus, the prevalence rate as determined by the Forced Response model with a standard error of 0.034159 and a 95% confidence interval of (0.02611, 0.14999) is estimated to be between 2.6% and 15.0%.

### Hair analysis

Among the available 154 hair samples, the presence of Mephedrone was found in six samples giving a 3.9% positive rate. As the quantity of substance potentially used and time of exposure is not known, it is plausible that the actual positive rate is higher than 3.9%. It is likely that the hair analysis would only capture 3 months preceding drug use and could not detect a single exposure, nor any use that might have taken place in the immediate two weeks preceding the sample collection during which the hair is still in the scalp. Thus this period is considered as a 'blind period' for hair analysis.

Combining these positive samples with known use from the questionnaire where respondents accidentally give away this information by either answering each question on the Single Sample Count/Unmatched list five or answered each question on the same individually, the prevalence rate rises to 5.7% (9/157). Two of the nine known positive cases overlap between analytical and questionnaire results.

### The simplified SSC algorithm

The fuzzy response SSC model is a new method and uses known population prevalence to estimate the proportion of affirmative answers to the sensitive question. As such, it is a simplified and more economical version of the Unmatched List Count using only one (experimental) sample. In order to avoid the need for a control sample (which inevitably leads to 50% loss of the sample), we embedded the target sensitive question into a set of four questions with 50-50 probability and benchmarked the sum of the number of observed 'yes' responses against the expected sum of the number of 'yes' responses for the four questions.

The benchmark questions were:

• My birthday is in the first 6 months (January - June) of the year.

• My house number is an even number.

• The last digit of my phone number is even

• My mother's birthday falls between July and December

The probability of a 'yes' answer to each of the four questions is therefore 50%, the expected average (sum of the number of 'yes' responses divided by the total number of responses) is two. Any upward deviation from this benchmark figure is the estimated proportion of 'yes' answers to the target question.

The target research question was:

• I have taken Mephedrone at least once in the previous three months

Respondents were instructed to indicate only the total number of their affirmative answers to the five questions without revealing which ones.

Based on the nature of the four non-sensitive questions, it was assumed that the population distribution for each question follows a binomial distribution, thus the distribution of the total number of 'yes' responses for non-sensitive questions is B(4*k, 0.5) where *k *is the sample size. In other word, the probability of an honest 'yes' response to each of the four non-sensitive questions is 50%. Assuming that there are equal numbers of 'yes' and 'no' responses to each of these four non-sensitive questions, it is possible to calculate the expected value of responses for the baseline non-sensitive questions:

Thus, if the probability distributions are exactly the same for all non-sensitive questions individually (assumed to be 0.5 in this case), the mean response for the four non-sensitive questions is expected to equal two, thus obtaining a mean response value greater than two is the indication of the estimated prevalence rate for the sensitive question. The prevalence rate estimation is calculated as:

where *d *is the estimated population distribution of the 'yes' answers to the sensitive question, *λ *is the observed number of 'yes' answers; and *n *is the sample size. The observed probability distribution of the number of 'yes' answers is shown in Table 3.

**Table 3:** Observed probability distribution of X = the number of 'yes' answers

X	Observed P(X)
0	0.063
1	0.270
2	0.376
3	0.215
4	0.068
5	0.008

The three-month prevalence rate and 95%CI for Mephedrone use, using the SSC method, was calculated as follows:

The observed number of 'yes' answers is derived from the sum of two random variables with distribution of B(4*237, 0.5) and B(237, *d*), where *d *is the population distribution of the sensitive key question and 237 was the number of respondents in the sample. The observed number of 'yes' answers in the sample was 469.

Whilst the distribution of the sum of these two random variables is unknown, we can make use of the normal approximation for a binomial distribution. A rule of thumb is that the normal approximation is applicable if *np *> 5 and n*(1-*p *) > 5, *d *> 0.021 and *d *< 0.979, where *n *and *p *are the distribution of the two binomial parameters. The normal approximation is derived as mean = *np *and variance = n*p*(1-*p*). Thus B(4*237, 0.5) is approximately the same as N(2*237, 237) and B(237, *d*) is N(237*d, 237*d*(1-*d*)). Since the maximum likelihood approximation of the mean of the normal distribution is the sample mean, 237*(*d*+2) = 469, hence *d *= -0.021097. Note that the estimated *d *is negative, since the observed number of 'yes' responses (469) is less than the expected number of 'yes' responses for the non-sensitive questions (474). This does *not *mean that the prevalence rate for Mephedrone is negative, only that the random fluctuations in the sample were too large and mask the expected upward bias in the number of observed 'yes' responses. We can nevertheless calculate the 95%CI for *d*, which is 469 ± *Z*(0.95)*√(237*(1+d*(1-*d*))), where *Z*(0.95) = 1.959964. Thus 95%CI is *d *± 0.12731334 = -0.021097 ± 0.12731334 = 0, 0.099634. Therefore the estimated prevalence rate for Mephedrone use is between 0 and 10.0%.

T-test statistics indicated that the mean score (1.9789, 95%CI 1.85, 2.11) obtained on the SSC did not differ significantly from 2, thus there was no evidence that the prevalence rate for Mephedrone use in the population would differ significantly from zero (t(236) = -0.3113, p = 0.7558, Cohen's d = 0.041). This non-significant test result can be explained by the relatively small sample size. Notably, the sample prevalence was estimated to be between 0 and 10%.

The above calculation holds if the probability distribution of answers to each baseline question is equal (e.g. 50/50 in all 4 cases), thus we can assume that the sum of the binomial distributions is also binomial. However, the sum of the binomials is not necessarily binomial if the probabilities vary among the questions. Therefore, in such cases the normal approximation is calculated individually for each question before the probabilities from the baseline questions are added together, as we know that the sum of the normal distributions also follows normal distribution.

### SSC algorithm taking the divergence from the 50/50 distribution into consideration

In order to test whether the estimation from the simplified SSC algorithm differs significantly from the estimation that takes the observed likely distribution for the 4 innocuous questions into consideration, we calculated *d *in a two-step process.

Firstly, we assumed that the probabilities of the innocuous binomial variables are not the same, so we estimated the probability distribution for each baseline question independently. In order to calculate the probabilities of the 4 innocuous binomial questions, we used the following datasets. For distribution of house and phone numbers, we used 7,500,000 UK residential data (usable dataset for house numbers: n = 6,859,957 and for phone numbers: n = 6,895,960) purchased from a commercial provider, whereas for birthdays, we used anonym datasets from two UK universities (n = 495,870 and n = 11,157). For the subsequent analysis, we used the large UK university dataset (n = 495,870) for birthdays. Details are presented in Table 4.

**Table 4:** Birthday distributions

	Frequency count	Probability	Frequency count	Probability
Birthday on/in^a^				
odd/even days	245,269	0.509872	235,771	0.490128
first half (up to and including the 15th)/second half of the month	239,157	0.497167	241,883	0.502833
first half/second half of the year	232,666	0.483673	248,374	0.516327
odd/even numbered months	242,683	0.504497	238,357	0.495503
				
Birthday on/in^b^				
odd/even days	253,438	0.511098	242,432	0.488902
first half (up to and including the 15th)/second half of the month	247,927	0.499984	247,943	0.500016
first half/second half of the year	247,447	0.499016	248,423	0.500984
Odd/even numbered months	251,226	0.506637	244,644	0.493363
				
Birthday on/in^c^				
odd/even days	5,739	0.514386	5,418	0.4856144
first half (up to and including the 15th)/second half of the month	5,562	0.498521	5,595	0.501479
first half/second half of the year	5,606	0.502465	5,551	0.497535
Odd/even numbered months	5,731	0.513669	5,426	0.486331

^a^US life insurance application data (n = 481,040)

^b^UK university registration data (n = 495,870)

^c^UK university registration data (n = 11,157)

House numbers (including apartment/flat number in the absence of house number) were split as 3,405,322 even (p = 0.4964057) and 3,454,635 (p = 0.5035943) odd numbers. 0.5 (t = -18.828, df = 6859956, p-value < 2.2e-16, 95% CI: 0.4960316, 0.4967799). Among the listed phone numbers, the last digit of the phone number was an even number in 3,429,497 cases (p = 0.4973197) with 3,466,463 last digits being an odd number (p = 0.5026803). The probability of a birthday falling on the first half of the year was p = 0.5004075 (247,447 cases) vs. 248,423 (p = 0.499016) birthdays registered for the second half of the year. Single sample t-test statistic testing H_0_: p = 0.5 for the 4 innocuous questions are as follows.

1. *My birthday is in the first 6 months *(January - June) of the year (t = -1.386, df = 495869, p = 0.1657; with estimated probability of 0.4990159 (95% CI = 0.4976242, 0.5004075)

2. *My house number is an even number *(t = -18.6633, df = 6952970, p < 0.001; with estimated probability of 0.49646115 (95% CI = 0.4960895, 0.4968328)

3. *The last digit of my phone number is even *(t = -14.077, df = 6895959, p < 0.001); with estimated probability of 0.4973197 (95% CI = 0.496946, 0.4976929)

4. *My mother's birthday falls between July and December *(t = 1.386, df = 495869, p = 0.165); with estimated probability of 0.5009841 (95% CI: 0.4995925, 0.5023758)

Therefore, we used these empirically derived probabilities to approximate normal distribution.

The number of 'yes' answers for the

1^st ^question is binomial, B(k, 0.4990159) → N(k*0.4990159, k*0.4990159*0.5009841)

2^nd ^question is binomial, B(k, 0.4964611) → N(k*0.4964611, k*0.4964611*0.5035389)

3^rd ^question is binomial, B(k, 0.4973197) → N(k*0.4973197, k*0.4973197*0.5026803)

4^th ^question is binomial, B(k, 0.5009841) → N(k*0.5009841, k*0.4990159*0.5009841)

Sensitive question is binomial, B(k, d) → N(k*d, k*d*(1-d))

Therefore, by adding these approximations together, the distribution of the 'yes' answers are

The Mephedrone dataset contained 469 'yes' answers from 237 respondents, therefore k = 237, and 237*(1.9937808+d) = 469, thus *d *= -0.0148779. The 95%CIs for the number of 'yes' answers with the above estimated mean and variance are439.0453 and 498.9547, thus *d *is between -0.1412 and 0.1115. Consequently, *d *(the estimated prevalence of Mephedrone use) is, indeed, between 0% and 11%, which is in keeping with the estimation we received using the simple algorithm with assumed p = 0.5 for 'yes' answers in all baseline non-sensitive questions. Therefore, applying the principles of Occam's razor, the simple algorithm should prevail.

## Triangulating the SSC with the FR and hair analysis

**Figure 1:**
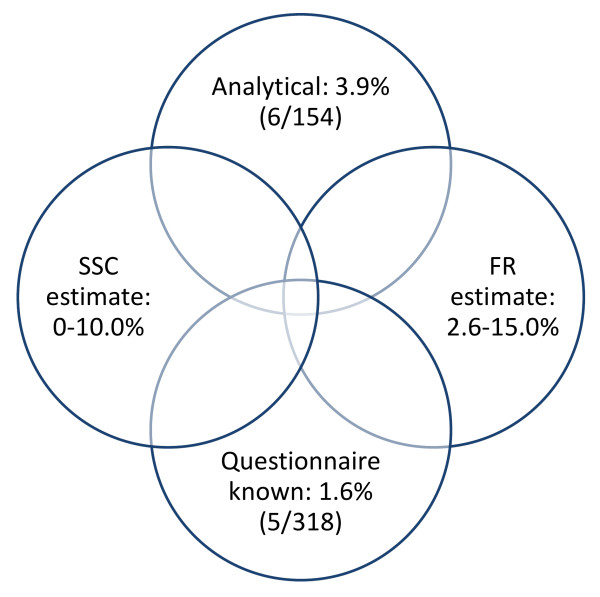
**Comparison of the two estimation methods (FR and SSC) hair sample results and limited information on Mephedrone use directly available from self-reports**.

The single most useful aspect of the hair analysis was to provide evidence that the sample prevalence of Mephedrone use was higher than zero. Figure 1 shows the combination of information available from the sample on Mephedrone use including an objective chemical analysis based on the presence of the drug in hair, accidental exposure via direct self-reports and two estimates representing two different indirect models. Combining these prevalence rates and estimates, we can conclude that the prevalence of Mephedrone use in the sample ranges between 5.7% and 15.0%. The two models yielded similar estimates with the FR up to 15% and the new SSC up to 10%. The close proximity of these estimates provides evidence that supports the validity of the new SSC model.

## Implementation

Practical issues relating to the indirect estimation methods are i) the chance of exposure, ii) minimum and optimal sample sizes required to achieve a desirable power, iii) efficiency and iv) potential to eliminate or detect noncompliance. This section discusses these in the context of implementing the SSC approach.

### Potential exposure

One notable drawback of the Single Sample Count model (as well as for the Unmatched List) is the scenario in which a respondent happens to have 'yes' answers to all innocuous questions and a 'yes' answers to the sensitive question. In this case, the respondent, if he/she answers truthfully, would reveal the information about the compromising behaviour. Note that the level of exposure in this situation becomes equivalent of the risk of exposure in an anonymous direct self-report.

This potential exposure situation can be mitigated by either increasing the number of innocuous questions (thus reducing the probability that such a scenario occurs (Table 5), or by offering an option of a new set of questions. Naturally, this latter option requires a bank of innocuous questions and only works in face-to-face interview settings or computer-assisted self-administration. Selecting the number of questions should take into consideration not only the probability but also the cognitive demand on respondents.

**Table 5:** The percentage of respondents potentially required to answer in a revealing way as the function of model design and prevalence rate of the sensitive question

Design	Innocuous	Sensitive^a^						
		**5%**	**10%**	**15%**	**20%**	**30%**	**40%**	**50%**
		
1 + 1	50.00	2.50	5.00	7.50	10.00	15.00	20.00	25.00
2 + 1	25.00	1.25	2.50	3.75	5.00	7.50	10.00	12.50
3 + 1	12.50	0.62	1.25	1.87	2.50	3.75	5.00	6.25
4 + 1	6.25	0.31	0.62	0.94	1.25	1.87	2.50	3.12
5 + 1	3.12	0.16	0.31	0.48	0.62	0.94	1.25	1.56
6 + 1	1.56	0.08	0.16	0.23	0.31	0.47	0.62	0.78
7 + 1	0.78	0.04	0.08	0.12	0.16	0.23	0.31	0.39
8 + 1	0.39	0.02	0.04	0.06	0.08	0.12	0.16	0.19

^a ^for illustration we assume that the compromising behaviour is proportionally distributed

**Figure 2:**
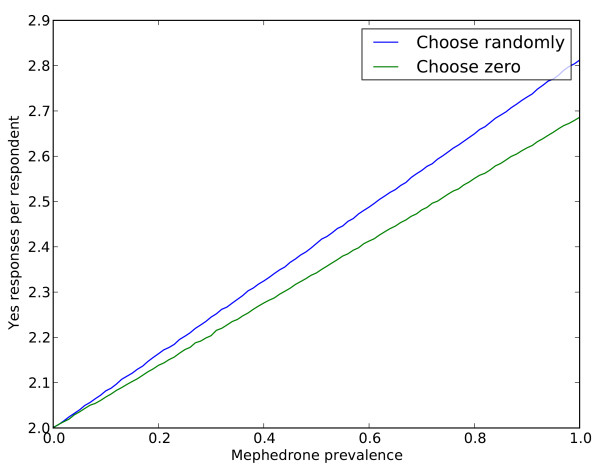
**Comparison of the simulated probability distributions under two scenarios: A) '0 & 5' combined and B) 'any other option' to avoid exposure (n = 1,000,000)**.

In cases where *d *is large, the potential exposure might be significantly high enough to consider alternative approaches. One example would be where answer options either combine 0 and 5 or allow respondent with answer '5' to select any answer options (0-4). Comparing the distribution of a hypothetical honest answer scenario with *d *= 0.2 prevalence rate for the sensitive question to the two proposed solutions using Kolmogorov-Smirnov's maximum divergence of the cumulative distribution function, no statistically meaningful preference was found between the two options (KS = 0.0125 for '0& 5'; and KS = 0.0125 for 'any option'). Using Root Mean Square (RMS) indicated a slight preference towards the 'any other' option (RMS = 0.0027 vs. RMS = 0.0035 for the '0 & 5'). Probabilities for the three scenarios are presented in Table 6. Simulations with 1 million data responses also showed very similar distributions (Figure 2). For simplicity, we assumed a 0.5 probability for each innocuous question. Given these results and taking practical issues into consideration, the combined 0 and 5 answer option is suggested for its relative simplicity. As one might expect, this solution to the '5-yes' problem affects the complexity of the computation to derive the estimated probability for the target sensitive question.

**Table 6:** Probability of answer distributions if i) questions are honestly answered, ii) 0 and 5 answers are combined and iii) respondents are instructed to select any response option; *d *= probability of doping

	Questions are honestly answered	0 and 5 answers are combined	Any other response options are selected
0	1/16 - d/16	1/16	1/16 - d/20
1	1/4 - 3d/16	1/4 - 3d/16	1/4 - 7d/40
2	3/8 - d/8	3/8 - d/8	3/8 - 9d/80
3	1/4 + d/8	1/4 + d/8	1/4 - 11d/80
4	1/16 + 3d/16	1/16 + 3/16d	1/16 + d/5
5	d/16		

### Required minimum sample size

**Figure 3:**
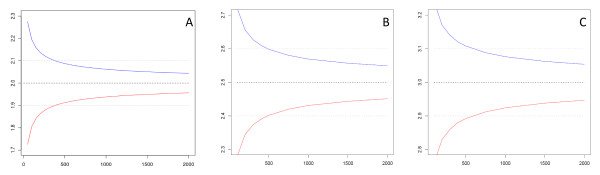
Function of sample size and 95%CI for (A) B(4*k, 05), (B) B(5*k, 05) and (C) B(6*k, 05).

Owing to the relatively small sample sizes, estimates using either the Forced Responses or the Single Sample Count method yielded negative values, making the lower bound of the 95%CI set to zero. The sample size required for the SSC model is chiefly determined by the sample size required to obtain a mean value for the four non-sensitive baseline questions to be as close as possible to two. Figure 3 shows that the bin width did not change significantly unless a significant increase in *n *is in place. Table 7 gives the exact values for the lower and upper 95%CI for selected sample sizes. From the practical point of view, the gain from increasing the sample by 500 is negligible compared to the potential cost of generating 500 samples. For comparison, 95%CIs are also calculated for 5 and 6 baseline questions, where the same logic applies as in the 4+1 model.

**Table 7:** 95%CI intervals for 4, 5 and 6 baseline question models when n = 100, 200, 300, 400, 500, 750, 1500 and 2000

Sample size	4 baseline questions B(4*k, 05)		5 baseline questions B(5*k, 05)		6 baseline questions B(6*k, 05)	
	**Lower**	**Upper**	**Lower**	**Upper**	**Lower**	**Upper**

100	1.800	2.200	2.280	2.720	2.760	3.240
200	1.860	2.140	2.345	2.655	2.830	3.170
300	1.887	2.113	2.373	2.627	2.860	3.140
400	1.903	2.098	2.390	2.610	2.880	3.120
500	1.912	2.088	2.402	2.598	2.892	3.108
750	1.928	2.072	2.420	2.580	2.912	3.088
1000	1.938	2.062	2.431	2.569	2.924	3.076
1500	1.949	2.051	2.443	2.557	2.938	3.062
2000	1.956	2.044	2.451	2.549	2.947	3.054

### Power analysis

The first four (non-sensitive) questions will be distributed B(n = 4, p = 0.5) while the 5^th ^question (sensitive question) will follow the Bernoulli distribution with a success probability of *p*, where *d *is the unknown prevalence rate. Let X = number of 'yes' answers out of the 5 questions, using the 4+1 SSC design.

Testing H_0_: μ = 2.0 vs. H_1_: μ > 2.0 with α = 0.05, the test statistic is calculated as:

Solve this equation for *n*:

With α = 0.05, the critical value *t *would be equal to 1.645. Substituting *t *= 1.645, *n *becomes a function of the effect size, Δ and standard deviation of X, *s*. We can calculate *s *from simulations using different prevalence levels. We will use *d *= 0.05, 0.10, 0.15,...0.50. This in turn will allow us to calculate the standard deviation of X, *s*.

From Table 8 below, it can be shown that when p = 0.15, *s *can estimated though 10,000 simulations to be 1.063. From this, recommended sample sizes can be developed. For example, to detect a significant effect size, Δ = 0.04 with α = 0.05 and *d *= 0.10, Table 8 shows that the simulated standard deviation would be s = 1.043 and the recommended sample size would be n = 1,839.

**Table 8:** Minimum sample sizes as the function of difference (denoted by Δ) for the 4 baseline question SSC model

	**Prevalence Rate**									
	0.05	0.1	0.15	0.2	0.25	0.3	0.35	0.4	0.45	0.5
	
	**Standard Deviation (s)**									
	1.022	1.043	1.063	1.080	1.092	1.104	1.112	1.118	1.118	1.121
	
Effect Size (Δ)	**Minimum Sample Size (n)**									
	
0.01	28247	29421	30566	31563	32251	32975	33479	33799	33823	33993
0.02	7062	7355	7641	7891	8063	8244	8370	8450	8456	8498
0.03	3139	3269	3396	3507	3583	3664	3720	3755	3758	3777
0.04	1765	1839	1910	1973	2016	2061	2092	2112	2114	2125
0.05	1130	1177	1223	1263	1290	1319	1339	1352	1353	1360
0.1	282	294	306	316	323	330	335	338	338	340
0.15	126	131	136	140	143	147	149	150	150	151
0.2	71	74	76	79	81	82	84	84	85	85
0.25	45	47	49	51	52	53	54	54	54	54
0.3	31	33	34	35	36	37	37	38	38	38
0.35	23	24	25	26	26	27	27	28	28	28
0.4	18	18	19	20	20	21	21	21	21	21
0.45	14	15	15	16	16	16	17	17	17	17
0.5	11	12	12	13	13	13	13	14	14	14

Mapping the information from Table 8 to Figure 3 and Table 7, it is easy to see the minimum required sample size is a direct function of the achieved bin width of the baseline questions. In practical terms, the required sample size ensures that the sample mean for the SSC is above the 95%CI for the baseline questions. The same logic applies for the models where the sensitive question is embedded in five or even the six innocuous questions. Table 9 displays the sample size values for the 5+1 SSC model.

**Table 9:** Minimum sample sizes as the function of difference (denoted by Δ) for the 5 baseline question SSC model

	**Prevalence Rate**									
	0.05	0.1	0.15	0.2	0.25	0.3	0.35	0.4	0.45	0.5
	
	**Standard Deviation (s)**									
	1.138	1.157	1.172	1.190	1.202	1.203	1.214	1.219	1.224	1.227
	
**Effect Size (Δ)**	**Minimum Sample Size(n)**									
	
0.01	35063	36243	37157	38294	39129	39155	39914	40224	40548	40713
0.02	8766	9061	9289	9574	9782	9789	9979	10056	10137	10178
0.03	3896	4027	4129	4255	4348	4351	4435	4469	4505	4524
0.04	2191	2265	2322	2393	2446	2447	2495	2514	2534	2545
0.05	1403	1450	1486	1532	1565	1566	1597	1609	1622	1629
0.1	351	362	372	383	391	392	399	402	405	407
0.15	156	161	165	170	174	174	177	179	180	181
0.2	88	91	93	96	98	98	100	101	101	102
0.25	56	58	59	61	63	63	64	64	65	65
0.3	39	40	41	43	43	44	44	45	45	45
0.35	29	30	30	31	32	32	33	33	33	33
0.4	22	23	23	24	24	24	25	25	25	25
0.45	17	18	18	19	19	19	20	20	20	20
0.5	14	14	15	15	16	16	16	16	16	16

Comparing the 4+1 model to the 5+1 model, the price that must be paid for the reduced chance of exposure is a slight increase in the required sample size. More importantly, however, this is in addition to the increased cognitive load on respondents which should be taken into consideration when designing the questionnaire.

In comparison, the minimum sample size for the FR is presented in Table 10 for SE {0.01, 0.02, 0.03, 0.04}, where we calculate a minimum sample size to form a confidence interval with varying levels of confidence. The conservative estimate of the minimum necessary sample size was calculated as follows:

**Table 10:** Minimum required sample size as a function of standard error (SE) with 95% confidence interval.

Standard error (SE)	Percentage points (1.96SE)	Minimum n
(0.05)	±0.0980	178
(0.04)	±0.0784	278
(0.03)	±0.0588	494
(0.02)	±0.0392	1112
(0.01)	±0.0196	4445

π_1 _= P(forced to say yes)

π_2 _= P(forced to answer honestly)

λ observed percent that responded 'yes'

Solve for *n*:

Maximize this equation by using λ = 0.5 and π_2 _= 3/4. Setting λ = 0.5 creates the maximum variance possible thus result in a conservative estimate establishing the necessary sample size assuming the worst case scenario.

Using a 95% confidence interval, we get:

Now use a standard error = {0.05, 0.04, 0.03, 0.02, 0.01}. Values are presented in Table 10.

For example, to obtain a 95% CI with width of 9.8 percentage points, n = 178 is required. To obtain a 95% CI with width of 1.96 percentage points, the required sample size rises to n = 4,445. This is comparable to the sample size required for the SSC to obtain a sufficiently narrow 95% CI for the innocuous questions (Table 7), which in turn, is very reassuring for the SSC as the FR model has been shown to be one of the most efficient model in terms of sample size with some 2.2 times of the direct question equivalent [17]. For example, Table 7 shows that with n = 400, the SE is 0.050 (n = 336 for this scenario in Table 10). Similarly, SSC n = 500, 1000 and 2000 give SE = 0.041, 0.032 and 0.023, respectively. These sample sizes map well onto those presented in Table 10 as n = 525, 934 and 2101, respectively. This congruence only holds for the 4+1 SSC design. As the number of the innocuous questions increases, so does the minimum required sample size. For example, reading from Table 7 (and in comparison to Table 10), we see that the sample required for SE ~ 0.03 is around 1,000 for the FR model and for the 4+1 SSC model, but reaching 1,500 for the 5+1 SSC with a further increase for the 6+1 SSC models. Thus the increase in sample size is the consequence of the increased security provided to respondents. Similarly, reducing the proportion in the FR model where honest answer is required results in increased security as well as in increased sample size. Notably, however, the large sample approximations of the proposed SSC method, along with other randomised response and non-random models, will provide reasonably close coverage for larger sample sizes, but may deviate from 95%CI for smaller sample sizes because of the discrete nature of the events.

### Efficiency

Unlike other RRT/NR models, the Singe Sample Count model uses every single response in the sample to estimate the prevalence rate for the sensitive question. As the population distribution is known *a priori*, there is no need to generate an independent sample from the same population to establish population prevalence. Thus the SSC model comes with no waste of any proportion of the sample. This aspect is unique among the RRT/NRM models.

### Non-compliance

The key driver for improving the random response and non-random models has been the hope that such techniques will be able to eliminate socially desirable responses. Social desirability (SD) is a known confounding factor in self reported research design, stemming from either the research tool or the person but equally resulting in dishonest responses [48,49].

Contrary to this desire, overwhelming evidence demonstrates that RRT/NRM are not cheating free [50,51]. Böckenholt et al. [51] used two separate methods, namely the Forced response and Kuk's [52] rather complicated card colour naming technique. Both demonstrated that accounting for non-compliance bias doubled the estimated prevalence. This finding is in-line with a medication non-adherence study that showed that almost half of the respondents did not follow the questionnaire instructions thus considerably distorting the prevalence rate without correcting for cheating [53]. This study used a variation of the forced response model linked to a rather low percentage when respondents have to answer honestly. The instructions were that if the respondent's father's birthday occurred in January or February then a truthful answer was requested, with a forced 'yes' for all other months. Therefore only 16.7% (2/12) of the respondents were asked to answer the sensitive question.

Self-protective no saying (SPN) is a known pattern in which respondents say 'no' without considering the instructions or truth. Considerable effort has been made to estimate the effect of dishonesty or correct for such effects [54-60]. Triggers for noncompliance could be the forced 'yes' answers in situations when respondents do not identify themselves with the discriminating behaviour; or complicated instructions which respondents are unable or unwilling to follow [51,61].

At this stage, we do not have data to ascertain what proportion of the responses on the SSC might have been affected by dishonest answering. Nonetheless, SSC does not offer an obvious self-protective response option as respondents who wish to deceive in their answers may simply chose entering zero, or any other number that is less than their true response would be. The somewhat higher estimate received using the SSC compared to the FR suggests that the SSC might be less affected by self-protective responding. Qualitative feedback received during data collection supports this assumption. Upon prompting for feedback in one group, respondents felt that they are more protected under the SSC model because as they phrased it: they "*didn't really have to answer the sensitive question*". This is, by design, was not the case in the FR model where depending on the outcome of the dice roll, 75% of the respondents were asked to answer the sensitive question.

### Potential innocuous questions

The SSC method builds on the innocuous question where the population distribution is assumed to be approximately close to 50/50. Such questions could be related to the last digits of a phone number, possibly house numbers or postcodes (even though these may vary from country to country), as well as birthdays. Selection of the most appropriate question must be informed by the research design, taking the target sample characteristics into consideration. Below, we present statistics derived from worldwide empirical data (n = 1,379), a publicly available dataset on birthdays (n = 481,040) and birthday data extracted from a UK university database (n = 495,870) to assist this process.

Empirical data were collected via Amazon Mechanical Turk in May-June 2011, with the Human Intelligence Tasks (HITs) made accessible worldwide to those with at least a 80% HITs acceptance rate [62]. The majority of the information was provided by people in India (59.2%), followed by the USA (28.4%), Canada (1.5%), Pakistan (1.1%) and the UK (1.0%). The remaining 51 countries contributed to a total of 8.8%. House numbers were odd numbers in 50.8%, whereas the last digits of the phone numbers were odd numbers in 48.6% of all records. Our results showed that more people prefer odd numbers for a lucky number (65.6%). The day of the birthday being odd occurred in 51.10% of the sample.

The publicly available birthday dataset was collected by Roy Murphy based on insurance policy applications to a Life Insurance Company between 1981 and 1994 http://www.panix.com/~murphy/bday.html and over 500 thousand birthdays captured in the internal information management system of two UK universities. The overall distribution of the birthdays in all three available datasets is remarkably similar to another database containing over 135 million records http://anybirthday.com/.

Using Roy Murphy's insurance application data, the results suggest that the 'first half vs. second half of the month' appears to give the closest split to 50/50, followed by the 'odd/even numbered month'. The analysis of two UK university population datasets of 495,870 and 11,157 birthdays provided further evidence that in the large dataset 'first half vs. second half of the month' lead to a closest split to 50/50, with the next closest distribution to 50/50 was the 'first vs. second half of the year' with the smaller dataset (n = 11,157) showing the opposite positions for the top two places. Frequency counts and probability distributions for birthdays falling on odd vs. even days and months; first vs. second half or the month and years, independently, are reported in Table 4.

## Discussion

The overarching advantage of both randomised response and non-random models is that they provide greater respondent anonymity protection as question responses cannot be traced to the individual. This anonymity also removes any ethical or legal obligation from the interviewer to act upon sensitive information disclosed to them as part of the research process.

Further advantages of the SSC method are:

• The model is simple to administer, offering a self-administration option without any sense of deception.

• The SSC model reduces the complexity in instructions and places low cognitive demands upon respondents.

• Unlike the FR model, SSC asks each respondent to answer, in a fuzzy way, the (sensitive) research question and hence improves the face validity of the research tool.

• Unlike other RRT/NR models, the SSC avoids a forced 'yes' response, which can be off-putting for people whose honest answer would normally be 'no' to the sensitive question. Also, respondents are not required to answer the sensitive question directly.

• In the SSC model, no obvious self protective strategy is present (e.g. self-protective 'no' saying), thus this approach can overcome the 'self protective no' bias.

The challenges with the SSC model arise from finding a suitable set of baseline questions where the population prevalence and distribution is known to be 50-50% and adequately addressing the chance of potential exposure. There is a small but existing chance that someone encounters a situation in which the answer would be revealing. This is not only a problem for the newly proposed Single Sample Count but also affects the classic Unmatched Count technique. The potential of exposure can be mitigated by either increasing the number of baseline questions to reduce the likelihood of having affirmative answers to all baseline questions or by offering different sets of baseline questions. This latter approach requires computerised administration or personal interviews.

This study would have further benefited from an increased sample size as confidence intervals were limited to the upper bounds. Further studies are required to improve the evidence base for testing the methodological validity and reliability. The sample size was confined by a number of specific criteria. The focus of the study was restricted to the use of a single substance (Mephedrone), which on the one hand held the advantage of requiring a single screening in hair, but on the other placed limits on the study for two reasons. Firstly, the population prevalence rate of illicit substance use was low because the study restricted itself to a specific substance. As the method for detecting Mephedrone in human hair is newly developed, it is not yet known what consumed quantity of the drug signals a positive analytical result, or how natural hair colour, sex or ethnicity, for instance, might affect the deposition of the drug in human hair. However, hair analysis may provide the ultimate gold standard for validating the SSC approach for substance use. If such is the case, then careful consideration must be accorded to the research design to ensure effective synergy between social, analytical and statistical approaches. It is important that the sensitive question considers the limitations imposed by the hair analysis. For example, some drugs deposit into hair with more ease than others and stay longer. Accidental or environmental exposure may be a contributing factor in explaining the presence of a given drug in hair samples. Hair analysis is normally not suitable to detect single or very recent (i.e. last two weeks) exposure - but if research remit requires knowledge of these aspects then urinalysis may be a viable alternative. The timeframe afforded by the selected biochemical analysis must be carefully matched in the question. Exploring these issues is beyond the scope of this paper.

Secondly, whilst the hair analysis component was useful to prove that the sample prevalence is larger than zero, its labour and costs implications placed limits on the sample size. We have compensated for this limitation with simulations to calculate the required sample sizes. The SSC model should also be tested investigating other discriminating behaviours with differing expected prevalence rates. If dishonest response patterns are known, prevalence estimation could incorporate a statistical correction component to account for this bias. Further refinement of the SSC model could include two variations where the sensitive question is positively (e.g. 'I have used drugs' or 'I do take my medications') or negatively framed (e.g. 'I have never used drugs' or 'I do not take my medications') to test whether giving confirmation, albeit indirect, of the desired or undesired behaviour has an effect on the results.

The research design could also benefit in some cases from the inclusion of a priming task to investigate whether or not the indirect approach and the additional protection afforded by the fuzzy response mode itself generates the maximum achievable admission of the discriminating behaviour. Alternatively, lie detector Implicit Associations Tests (e.g. [63,64]) could be combined with the SSC models for contrasting and comparing prevalence rates obtained via different methods from the same sample.

## Conclusion

The major advantage of the Single Sample Count method over other models such as the Forced Response model is rooted in its simplicity, equal face validity for each respondent, simple calculations and maximum use of the data. This elegantly simple, quick and cost effective method can be successfully employed in public health research aiming to establish the epidemiology of potentially compromising behaviours. Notwithstanding, this approach, akin to other randomised and non-random models, is suitable to establish group level prevalence.

## Conflicting interests

The authors declare that they have no competing interests.

## Authors' contributions

AP and DPN initiated the project. AP devised the study, contributed to analyzing the data and prepared the first draft of the manuscript. PC, HT and CA collected the data and contributed to the final draft of the manuscript. TN, MS and JS analysed the survey data and provided the additional statistical information. SH, ND, DPN and JB developed the method for and conducted the hair analysis; and prepared the relevant section of the manuscript. All authors have contributed to drafting the paper; read and approved the final version of the manuscript.
